# Complexity of temperature dependence in methanogenic microbial environments

**DOI:** 10.3389/fmicb.2023.1232946

**Published:** 2023-07-06

**Authors:** Ralf Conrad

**Affiliations:** Max Planck Institute for Terrestrial Microbiology, Marburg, Germany

**Keywords:** methanogenesis, activation energy, temperature optima, thermodynamic, microbial community

## Abstract

There is virtually no environmental process that is not dependent on temperature. This includes the microbial processes that result in the production of CH_4_, an important greenhouse gas. Microbial CH_4_ production is the result of a combination of many different microorganisms and microbial processes, which together achieve the mineralization of organic matter to CO_2_ and CH_4_. Temperature dependence applies to each individual step and each individual microbe. This review will discuss the different aspects of temperature dependence including temperature affecting the kinetics and thermodynamics of the various microbial processes, affecting the pathways of organic matter degradation and CH_4_ production, and affecting the composition of the microbial communities involved. For example, it was found that increasing temperature results in a change of the methanogenic pathway with increasing contribution from mainly acetate to mainly H_2_/CO_2_ as immediate CH_4_ precursor, and with replacement of aceticlastic methanogenic archaea by thermophilic syntrophic acetate-oxidizing bacteria plus thermophilic hydrogenotrophic methanogenic archaea. This shift is consistent with reaction energetics, but it is not obligatory, since high temperature environments exist in which acetate is consumed by thermophilic aceticlastic archaea. Many studies have shown that CH_4_ production rates increase with temperature displaying a temperature optimum and a characteristic apparent activation energy (*E_a_*). Interestingly, CH_4_ release from defined microbial cultures, from environmental samples and from wetland field sites all show similar *E_a_* values around 100 kJ mol^−1^ indicating that CH_4_ production rates are limited by the methanogenic archaea rather than by hydrolysis of organic matter. Hence, the final rather than the initial step controls the methanogenic degradation of organic matter, which apparently is rarely in steady state.

## Introduction

There is virtually no microbial activity that would not be regulated by temperature (Wiegel, 1990). Biological activity is based on chemical reactions. Chemical reactions are controlled by temperature and so is biological activity. Therefore, temperature dependence of biological activity follows the same physical principles as chemical reactions do. In fact, it is predominantly enzyme-based biochemical reactions, which control biological activity. Therefore, biological activity always exhibits a temperature optimum, beyond which enzymes become inactivated (Radmer and Kok, 1979), while purely chemical reactions may tolerate much higher temperatures.

Microbial life is complex. Microorganisms contain many different enzymes, which may react differently upon temperature changes. Microbial populations consist of many different individual microorganisms, each possibly slightly different in its response to temperature. In nature, microbial communities consist of many different microbial populations with different physiologies and life styles and thus, with potentially different features of temperature dependence. Therefore, it is by principle very complex how environmental microbial communities will react to temperature changes and it is hard to make any predictions.

Nevertheless, it is worthwhile to review the literature on temperature dependence of microbial activity to see whether there are any guiding principles. In the following I will primarily (but not exclusively) focus on methanogenic microbial communities living in anoxic environments such as flooded rice fields and aquatic sediments, whose temperature characteristics have frequently been studied over the last 40–50 years. A previous mini-review on this subject (Conrad, 2008) will be updated and expanded. The temperature dependence of methanogenic communities is of particular interest, since methane is an important greenhouse gas, which is partially responsible for past and present climate change and will in turn be affected by the global temperature increase in many respects (Kirschke et al., 2013).

## Temperature dependence of methane-producing microbial populations

Methane is produced by methanogenic archaea, which convert simple substrates like acetate, H_2_ + CO_2_, formate, trimethylamine, dimethylsulfide, and methanol to CH_4_. The most common methanogenic substrates in nature are acetate and H_2_ + CO_2_ (Conrad, 2020). The rates of CH_4_ production may be described by Michaelis–Menten kinetics, which account not only for the maximal possible rate, *V_max_*, but also for the effect of the substrate concentration, S, i.e., v = *V_max_* S/(*K_m_* + S). The *K_m_* value is the substrate concentration at *V_max_*/2. In this way, metabolic rates are modulated by the substrate availability. Both *V_max_* and *K_m_* of enzymatic reactions are expected to increase with increasing temperature (Scopes, 1995). Microbial growth rates, μ, are analogously modulated by substrate concentration using the Monod equation, i.e., μ = *μ_max_* S/(*K_S_* + S), where *K_S_* is the substrate concentration at *μ_max_*/2. Note, however, that *K_m_* and *K_S_* are different values with a different physiological meaning and relevance.

The rates of CH_4_ production increase with increasing temperature. The increase in rate (v) can be described by the Arrhenius equation, i.e., v = A exp {−*E_a_*/(RT)} with A = Arrhenius constant and *E_a_* = apparent activation energy. In a microbial cell, e.g., a methanogenic archaeon, CH_4_ is produced as the end product of a catabolic reaction chain, in which the methanogenic substrate is converted to CH_4_ (Thauer et al., 2008). The rate-limiting step is usually not the reaction producing the CH_4_, but the first enzymatic step in the entire process chain, since otherwise process intermediates would accumulate (Heinrich and Schuster, 1996). It is generally assumed that the transport of the substrate over the cellular membrane is the step, which limits catabolism and growth (Button, 1985). The cell-specific affinity, which is equal to *V_max_*/*K_m_*, is decisive for substrate uptake and catabolism. It has been argued that substrates with active transport over the cellular membrane (e.g., nitrate) face a cell-specific affinity that increases with temperature, whereas substrates without active transport (e.g., ammonia) face a temperature independent cell-specific affinity (Nedwell, 1999). The methanogenic substrates H_2_ and CO_2_ display no active transport. However, determination of Michaelis–Menten parameters of process kinetics in methanogenic microbes showed that both *V_max_* and *K_m_* exhibit temperature dependence (Westermann et al., 1989; Kotsyurbenko et al., 2001). The increasing *V_max_* is compensated by the also increasing *K_m_* thus resulting in fairly constant cell-specific affinities (*V_max_*/*K_m_*), such as predicted by Nedwell (1999). Therefore, we have to assume that CH_4_ production rates are not limited by the substrate transport of substrates, but by one of the subsequent enzymatic reaction steps. This limiting enzyme reaction may be the same as that defining the kinetic isotope effect of CH_4_ production (Games et al., 1978). The kinetic isotope effect, which by itself is not influenced by temperature (Penger et al., 2014), is assumed to be influenced by the first irreversible enzymatic steps, which result in a “commitment of reaction” (Northrop, 1981; Thullner et al., 2013). For aceticlastic methanogens it is the activation of acetate by either acetate kinase or acetyl CoA synthase, resulting in stronger isotope effect in *Methanosarcina* versus *Methanothrix* (*Methanosaeta*) species, respectively (Penning et al., 2006; Goevert and Conrad, 2009). These two methanogenic genera typically also display different *μ*_*max*,_ *V_max_*, *K_S_*, *K_m_* and thresholds for acetate by having either of the two acetate-activating enzyme systems (Jetten et al., 1992). It is likely that these enzyme systems constitute the rate limiting steps for aceticlastic CH_4_ production displaying temperature dependence.

In any case, the temperature characteristic described by *E_a_* is that of the rate-limiting step of a particular methanogenic archaeon and a particular substrate. The *E_a_* values of different methanogenic archaea are typically 106 kJ mol^−1^ (89–122 kJ mol^−1^). These data are from a meta-analysis of literature data, based on 33 different strains of growing or non-growing methanogenic archaea (Yvon-Durocher et al., 2014). These *E_a_* values are relatively high when compared to respiration (Yvon-Durocher et al., 2012), photosynthesis (Allen et al., 2005) and hydrolysis of organic matter (Middelburg et al., 1996; Weston and Joye, 2005).

## Temperature dependence of methane-producing microbial communities

**Figure 1:**
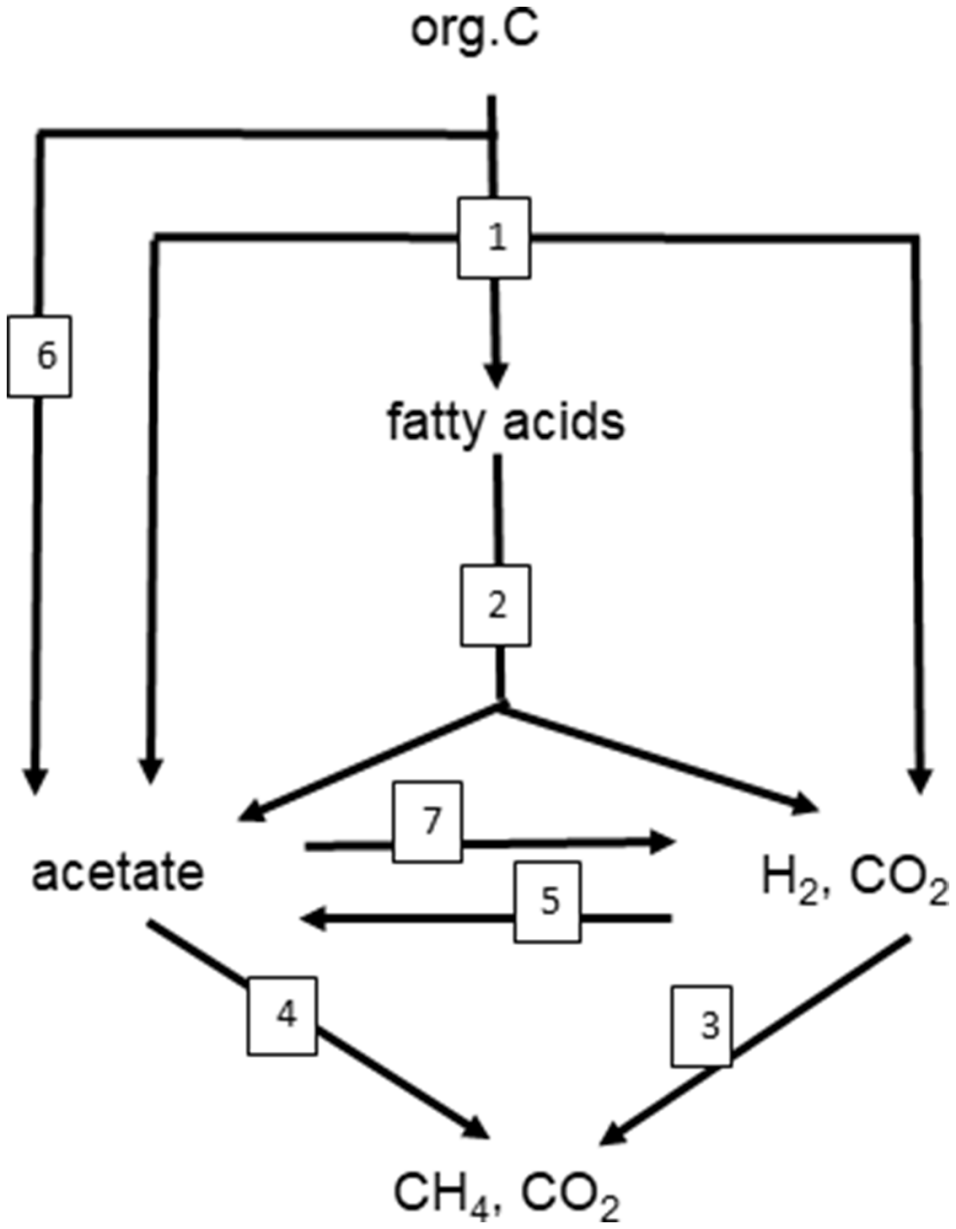
Scheme of the anaerobic degradation of organic matter (e.g., cellulose) to CH_4_ and CO_2_. The degradation involves the following functions indicated by numbers: (1) hydrolysis of polymeric organic matter to monomers, followed by fermentation of the monomers to short-chain fatty acids (e.g., lactate, propionate), acetate and H_2_, CO_2_; (2) syntrophic conversion of short-chain fatty acids to acetate, H_2_, CO_2_; (3) methanogenic conversion of H_2_, CO_2_ to CH_4_ (hydrogenotrophic methanogenesis); (4) methanogenic conversion of acetate to CH_4_ (aceticlastic methanogenesis); (5) conversion of H_2_, CO_2_ to acetate (chemolithotrophic homoacetogenesis); (6) fermentation of monomers to acetate only (heterotrophic homoacetogenesis); (7) syntrophic oxidation of acetate to H_2_, CO_2_. Stoichiometries and thermodynamic parameters of examples for the numbered reactions are shown in Table 1.

**Figure 2:**
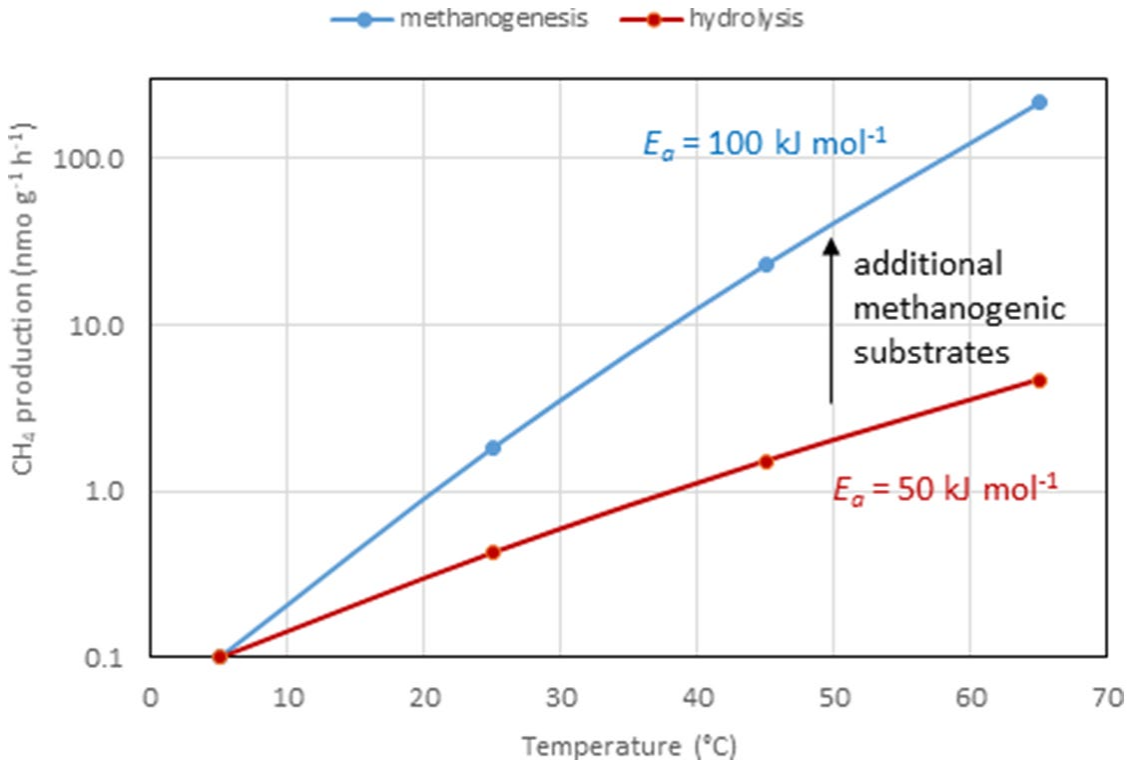
Temperature dependence of rates of CH_4_ production and hydrolysis of organic matter by assuming *E_a_* values of 100 and 50 kJ mole^−1^, respectively.

A similar range of *E_a_* values was obtained from 47 different methanogenic microbial communities in paddy soils, wetlands and aquatic sediments, i.e., 89 kJ mol^−1^ (79–99 kJ mol^−1^; Yvon-Durocher et al., 2014). In these environmental samples CH_4_ is produced as the final step in a complex microbial community that converts organic matter to CH_4_ and CO_2_. Polysaccharides, a common form of organic matter, are hydrolyzed to sugars, which are then fermented by bacteria to fatty acids, alcohols and other small compounds. These are then further fermented to acetate, H_2_ and CO_2_ by bacteria living in syntrophic association with methanogenic archaea, which produce CH_4_ (Zinder, 1993; Schink and Stams, 2013; Figure 1). If the system is in steady state, the first step, i.e., hydrolysis of organic matter, should be the rate-limiting step of CH_4_ production, and all the subsequent reactions should be substrate-limited (Nedwell, 1984). However, this seems to be rarely the case. Thus, CH_4_ production increases if the methanogenic substrates acetate and H_2_ are added showing that temperature limits substrate supply (Westermann, 1993; Nozhevnikova et al., 1997). Addition of polysaccharides or sugars results in enhancement of fermentation reactions and the production of acetate and H_2_, and consequently also stimulates CH_4_ production (Kotsyurbenko et al., 1993; Chin et al., 1998). Stimulation of CH_4_ production by substrate addition usually is stronger at high than at low temperatures, so that *E_a_* values increase. For example, addition of excess H_2_ to methanogenic paddy soil increased the *E_a_* values from about 70 to 91 kJ mol^−1^ (Conrad et al., 1987). This response is plausible if the *E_a_* values of the terminal CH_4_ production step are larger than those of the preceding processes such as polysaccharide hydrolysis or fermentative H_2_ production (Figure 2). In experiments with marine sediments, *E_a_* values of hydrolysis/fermentation indeed are on the average only 49 kJ mol^−1^ (Weston and Joye, 2005). In other marine sediments, however, the range of *E_a_* values for hydrolytic processes extends from 54 to 125 kJ mol^−1^ (Middelburg et al., 1996). After submergence of rice field soils, degradation of labile organic matter usually increases the production of the methanogenic substrates acetate and H_2_ over several days before CH_4_ production eventually enters quasi steady state conditions with much lower rates. Values of *E_a_* are typically much lower (more than three times) during steady state than during the phase of excess substrate (Yao and Conrad, 2000). Hence, the relatively narrow range and the relatively high values of *E_a_* for CH_4_ production obtained by the meta-analysis of Yvon-Durocher et al. (2014) indicate that the microbial communities are not in steady state with the hydrolysis of organic matter. This is even more so, since the mean value (89 kJ mol^−1^) is close to that of defined methanogenic populations (106 kJ mol^−1^) suggesting that CH_4_ production by methanogenic archaea is the rate-limiting step also when organic matter is the primary substrate (Hoehler and Alperin, 2014). This observation suggests that the methanogenic archaea in the complex communities are not limited by supply of their substrates (i.e., acetate, H_2_ + CO_2_) when temperature increases. An alternative explanation is that the hydrolysis of organic matter, as the initial rate-limiting step under steady state conditions, has a similar *E_a_* value as the CH_4_ production by methanogenic archaea (Hoehler and Alperin, 2014). Note that literature data show a rather large range of *E_a_* values from 49 to 125 kJ mol^−1^ (Middelburg et al., 1996; Weston and Joye, 2005). Also note, that the different hydrolytic enzymes may be differently affected by temperature (Fey and Conrad, 2003; Arnosti and Joergensen, 2006) and that the effective composition of organic matter, which is converted to CH_4_, changes with incubation time, resulting in a change of the pathway of CH_4_ formation (Ji et al., 2018).

## Temperature dependence of methane-producing ecosystems

Meta-analysis of 127 sites of methanogenic ecosystems (marshes, rice fields, peatlands) shows a narrow range of *E_a_* values, i.e., 92 kJ mol^−1^ (83–103 kJ mol^−1^; Yvon-Durocher et al., 2014). These values are similar to those found in methanogenic communities and in methanogenic populations, again suggesting that the temperature dependence of CH_4_-emitting ecosystems is the same as that of methanogenic microbial cultures rather than that of enzymes hydrolyzing organic matter. The *E_a_* values in one Italian rice field exhibited a broad range from 50 to 450 kJ mol^−1^ (Schütz et al., 1990). The meta analysis of various ecosystems also showed a broad frequency distribution of *E_a_* values (Yvon-Durocher et al., 2014). Nevertheless, the mean *E_a_* values of CH_4_-emitting ecosystems and CH_4_-producing methanogenic microbial cultures were statistically indistinguishable. This is a remarkable observation, especially since the *E_a_* values related to CH_4_ emission are relatively high. They are much higher than those related to respiration (Yvon-Durocher et al., 2012), photosynthesis (Allen et al., 2005) or to the CO_2_ emission rates observed in the same meta analysis (Yvon-Durocher et al., 2014).

In order to appreciate the coincidence of *E_a_* values of methanogenic archaeal populations with those of entire ecosystems, one should realize that at the level of ecosystems the temperature changes are recorded as daily rhythms and seasonal variations. In addition to the temperature effects, ecosystems are exposed to many other control variables, such as variations in substrate supply [e.g., root exudations in vegetated soil and sediment (Watanabe et al., 1999)], consumption of CH_4_ at the oxic sediment surface or the rhizosphere (Schütz et al., 1989), or simply by temperature gradients within the ecosystem (sediment depth; Schütz et al., 1990). Usually the microbial community is also exposed to various potential oxidants such as nitrate, ferric iron, and sulfate (Zehnder and Stumm, 1988). These oxidants allow the oxidative catabolism of the organic substrates to CO_2_ in the community thus suppressing CH_4_ production. Production of CH_4_ is suppressed, since the process kinetics and thermodynamics of microorganisms reducing nitrate, ferric iron, or sulfate allow to outcompete methanogenic archaea for their substrates H_2_ and acetate (Cord-Ruwisch et al., 1988; Lovley and Goodwin, 1988). However, CH_4_ production is initiated as soon as these oxidants are depleted. Since these oxidation processes are temperature dependent in a similar way as the processes leading to CH_4_ production, the different phases of sequential reduction of oxidants can strongly affect the *E_a_* values until quasi steady state is reached (Nozhevnikova et al., 1997; VanBodegom and Stams, 1999; vanHulzen et al., 1999; Yao and Conrad, 2000). In summary, CH_4_ emissions from ecosystems exhibit a temperature dependence that is hardly consistent with the assumption of being in steady state with organic matter degradation in the soil or sediment. Instead, the CH_4_-producing methanogens must be supplied with additional substrate. In rice fields and vegetated wetlands it may be photosynthetically produced organic substrates, which are supplied as root exudation. In fact, this source of organic carbon seems to be responsible for more than 50% of total CH_4_ emission (Watanabe et al., 1999; Tokida et al., 2011; Yuan et al., 2012). In lake sediments, fresh organic carbon can be supplied by sedimentation of algae (Schulz and Conrad, 1995; Schwarz et al., 2008). Furthermore, fresh organic matter has a short lifetime in paddy soil so that 80–90% is degraded within 1 year (Neue and Scharpenseel, 1987). The composition of the degradable organic matter in paddy soil changes over time, thus causing a change in the degradation pathway to CH_4_ (Ji et al., 2018). Seasonal changes in the methanogenic pathway are commonly observed in rice field ecosystems (Bilek et al., 1999; Krüger et al., 2002; Zhang et al., 2013). Hence, it is not surprising that hydrolysis of organic matter is usually not the rate-limiting step of CH_4_ production in the environment, but other processes forming methane precursors.

The temperature dependence of CH_4_ production is similar in tropical versus boreal lake sediments (Marotta et al., 2014). Since sediments of low latitude versus high latitude lakes are much warmer, greenhouse gas production is also much higher in those regions despite their much smaller lake area, and they will also respond more strongly to global warming because of the exponential effect of *E_a_*.

## Temperature optimum of methane production

Production of CH_4_ exhibits a temperature optimum beyond which the existing microorganisms and their enzyme systems become inactivated or beyond which methanogenic life does not exist. Such a temperature optimum can be modelled within the framework of the Arrhenius theory by a temperature-dependent change in the heat capacity for enzyme catalysis (Hobbs et al., 2013; Schipper et al., 2014). Whenever methanogenic environmental samples have been incubated at different temperatures, the highest CH_4_ production rates have generally been observed at mesophilic temperatures around 30°C (Zeikus and Winfrey, 1976; Conrad et al., 1989; Nozhevnikova et al., 1997; Fey and Conrad, 2003; Metje and Frenzel, 2007; Blake et al., 2015). Occasionally, a second optimum has been observed at thermophilic temperatures of 50–70°C (Nozhevnikova et al., 1997; Fey et al., 2001). It is remarkable that the temperature optima are much higher than the average *in-situ* temperatures. This is especially notable for profundal lake sediments, which are at permanently low 4°C, while temperature optima are around 30°C. The display of temperature optima higher than *in-situ* indicates the existence of mesophilic microbes that rapidly respond to a temperature increase, thus outcompeting the microorganisms active at *in-situ* temperature. These mesophilic microorganisms must cover the entire microbial community responsible for CH_4_ production, including hydrolytic and fermentative bacteria. The difference between *in-situ* temperature and temperature optimum is not restricted to methanogenesis, but seems to be a general feature of sediment metabolism (Yayanos, 1986; Arnosti et al., 1998).

Nevertheless, microbial and enzymatic activity is also found at the low *in-situ* temperatures (Schulz and Conrad, 1996; Arnosti and Joergensen, 2003; Metje and Frenzel, 2007; Kolton et al., 2019). This can be interpreted either as psychotolerance of mesophilic microorganisms or as the existence of a hidden psychrophilic microbial community. Psychotolerance seems to be a widely occurring phenomenon (Nozhevnikova et al., 2001, 2003; Saunders et al., 2003; Kotsyurbenko, 2005). However, reports of psychrophilic methanogenic archaea, which are not only tolerant but are especially adapted to life at low temperatures are not very common (Franzmann et al., 1997; Simankova et al., 2001, 2003; Cavicchioli, 2006; Parshina et al., 2014; Zhou et al., 2014). This is in contrast to the abundance of hyperthermophilic methanogenic species (Stetter, 2002), indicating that methanogenic archaea potentially adapt better to high than to low temperatures. This preference may be explained by the specificities of cellular characteristics in *Archaea* versus *Bacteria* (Cavicchioli, 2006; Valentine, 2007). For example, the microbial cell membranes of *Archaea* and *Bacteria* are ether lipid monolayers and fatty acid ester bilayers, respectively. The temperature dependence of membrane rigidity and flexibility is different for the two types of lipid membranes (Koga, 2012; Siliakus et al., 2017), thus affecting microbial adaptation. Since different microbial populations may display different temperature adaptations, the optimum temperature can be different for different physiological groups and different biogeochemical pathways, e.g., hydrogenotrophic and acetotrophic methanogenesis (Svensson, 1984; Schulz et al., 1997). In fact, the pathway of CH_4_ production is strongly affected by temperature (see below).

## Temperature dependence of process thermodynamics

**Figure 3:**
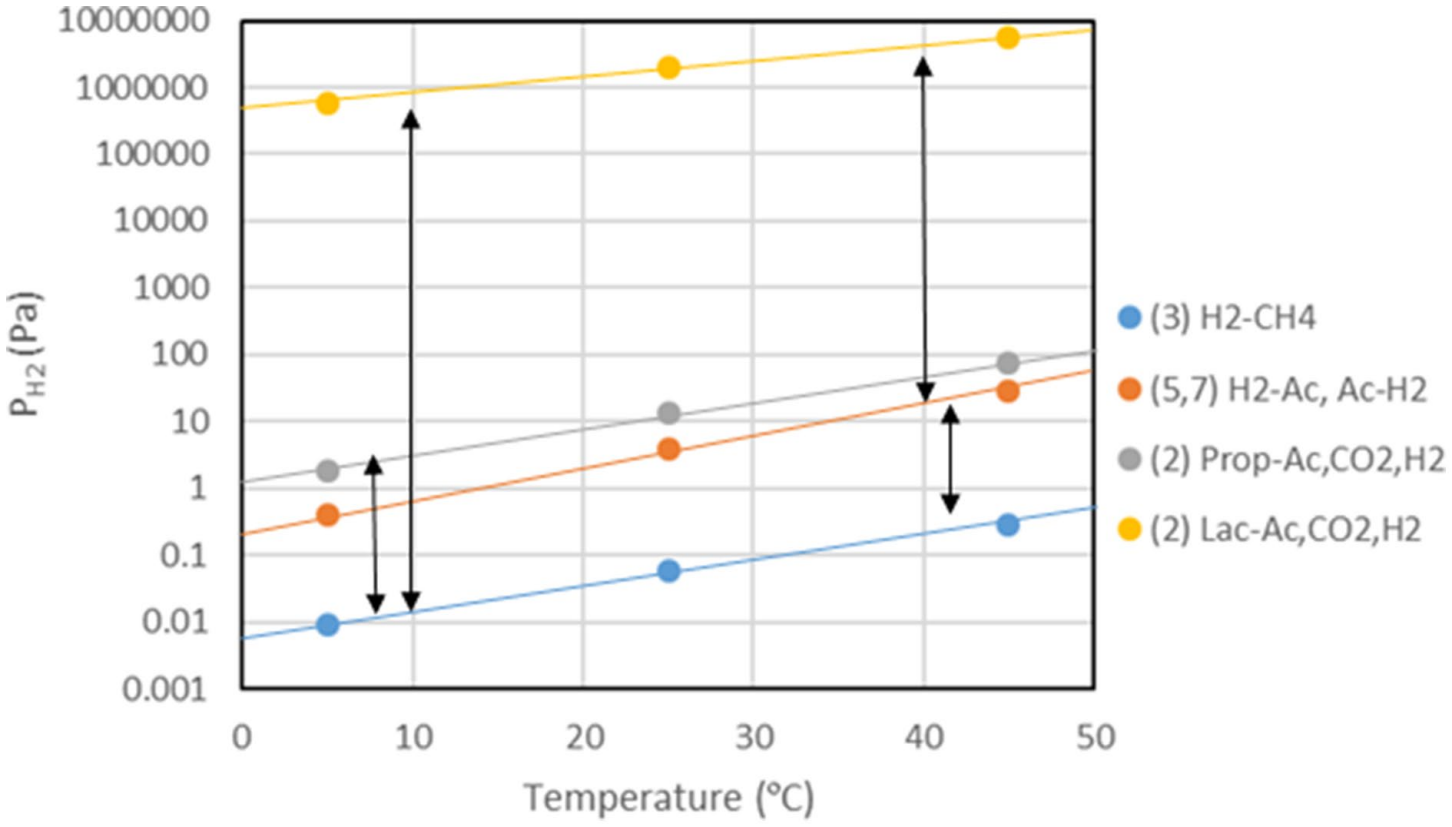
Temperature dependence of H_2_ thresholds of H_2_-producing and H_2_-consuming processes. The double arrows indicate the window of H_2_ partial pressures in which syntrophic interaction between the H_2_-producing and H_2_-consuming processes is possible. The H_2_-thresholds were calculated for ΔG’_T_ = 0 using the Nernst equation with CH_4_ = 0.001 bar, CO_2_ = 0.1 bar, and lactate, propionate and acetate = 1 mM. The numbers indicate the processes listed in Table 1.

Besides enzyme kinetics, temperature also affects the thermodynamics of methanogenic processes. The free enthalpy (Gibbs free energy) change of a reaction is given by the changes of the enthalpy and the entropy, i.e., ΔG = ΔH – TΔS. For standard conditions (1 atm, 1 M, 298°K, pH = 0) values of ΔG^o^, ΔH^o^, and ΔS^o^ for reactions important in CH_4_ production pathways can be calculated from tabulated values (e.g., Stumm and Morgan, 1996; Table 1). Values of ΔG^o^´ give the standard free enthalpy at pH 7 if n protons are produced or consumed in the reaction, i.e., ΔG^o^´ = ΔG^o^ ± nRT ln(10^−7^), which is −39.94 kJ mol^−1^ protons produced. Temperature dependence of ΔG^o^ is basically a function of the magnitude of the process entropies (ΔS^o^) under standard conditions. The actual ΔG at the given concentrations of reactants and products can be calculated from the Nernst equation, which is also temperature-dependent, i.e., ΔG = ΔG^o^ + RT ln(P/S), where P are the products and S the reactants. P and S represent the product of the activities or partial pressures of the individual products and reactants, respectively. At dilute solutions activities may be replaced by concentrations. The lowest possible concentrations of the reactants are those when the actual ΔG = 0, i.e., the threshold. For example, hydrogenotrophic methanogenesis proceeds until the threshold concentration (partial pressure) of H_2_ is reached. Below the threshold H_2_ can no longer be consumed and CH_4_ production stops (Figure 3). On the other hand, there is also an upper threshold of H_2_, which may not be surpassed to allow the syntrophic degradation of compounds such as lactate, propionate, or acetate (Figure 3). The degradation of such compounds is endergonic under standard conditions, but becomes exergonic if the H_2_ partial pressures are sufficiently low (McInerney et al., 2008; Schink and Stams, 2013). The thresholds of H_2_ consumption by methanogenesis or sulfate reduction are usually a bit higher than predicted by ΔG = 0 (Yao and Conrad, 1999; Hoehler et al., 2001) and those of H_2_ production by syntrophic fatty acid degradation are usually a bit lower (Scholten and Conrad, 2000; Yao and Conrad, 2001). The deviation is on the order of 3–6 kJ mol^−1^ H_2_. It is probably due to the fact that the microorganisms require a minimum of free enthalpy to allow the synthesis of 1/4–1/3 ATP (Thauer and Morris, 1984; Schink, 1997; Lever et al., 2015). The H_2_ threshold has also been explained by models, e.g., by a combination of the thermodynamic equilibrium constant (K = exp {−ΔG^o^/(RT)}) with the Michaelis–Menten equation (Hoh and Cord-Ruwisch, 1996), or by including the maintenance energy requirements of the microorganisms (Hoehler, 2004).

**Table 1:** Entropies (ΔS^0^) and free enthalpies (ΔG^0^) under standard conditions (1 bar, 1 M, 298°K, pH = 0) and at pH = 7 (ΔG^0^´) for different reactions involved in the degradation of hexose to CH_4_.

Reaction	ΔS^0^	ΔG^0^	ΔG^0^´
	kJ K^−1^	kJ	kJ
1) C_6_H_12_O_6_ → 2CH_3_CHOHCOO^−^ + 2H^+^	+0.165	−125	−205
1) CH_3_CHOHCOO^−^ → 2/3CH_3_CH_2_COO^−^ + 1/3CH_3_COO^−^ + 1/3CO_2_ + 1/3H_2_O	−0.004	−53	−53
2) CH_3_CHOHCOO^−^ + H_2_O → CH_3_COO^−^ + CO_2_ + H_2_	+0.306	−9	−9
2) CH_3_CH_2_COO^−^ + 2H_2_O → CH_3_COO^−^ + CO_2_ + 3H_2_	+0.447	+72	+72
3) 4H_2_ + CO_2_ → CH_4_ + 2H_2_O	−0.410	−131	−131
4) CH_3_COO^−^ + H^+^ → CH_4_ + CO_2_	+0.315	−76	−36
5) 4H_2_ + 2CO_2_ → CH_3_COO^−^ + H^+^ + 2H_2_O	−0.723	−55	−95
6) C_6_H_12_O_6_ → 3CH_3_COO^−^ + 3H^+^	+0.041	−197	−317
7) CH_3_COO^−^ + H^+^ + 2H_2_O → 4H_2_ + 2CO_2_	+0.723	+55	+95

The ΔG^0^_T_ at a particular temperature T is given by ΔG^0^_T_ = ΔG^0^ − ΔS^0^ (T – 298). The numbers of the reactions correspond to those in Figure 1.

Thresholds of H_2_ partial pressures are a function of the standard free enthalpies of anaerobic H_2_-consuming processes, and generally decrease with the useful energy (more negative ΔG^o^). Hence, the H_2_ threshold is for example lower in hydrogenotrophic ferric iron reducers < sulfate reducers < methanogens < homoacetogens. This has been found in microbial populations (Lovley, 1985; Cord-Ruwisch et al., 1988; Seitz et al., 1990; Caccavo et al., 1992) and in methanogenic environments (Conrad et al., 1986; Lovley and Goodwin, 1988; Hoehler et al., 1998). In the environment, CH_4_ production from H_2_/CO_2_ only operates, if the H_2_ partial pressures are higher than the threshold (Rothfuss and Conrad, 1993; Yao and Conrad, 1999; Hoehler et al., 2001). By contrast, generation of H_2_ from ethanol or fatty acids only operates, if H_2_ partial pressures are lower than the threshold (Seitz et al., 1990; Westermann, 1994; Scholten and Conrad, 2000; Yao and Conrad, 2001). Hence, metabolism and growth of both H_2_-producing and H_2_-consuming microorganisms is thermodynamically restricted in opposite ways. The satisfaction of the upper and the lower thresholds creates a window of permissive H_2_ partial pressures. Only within this window is the syntrophic conversion of ethanol, fatty acids and aromatic compounds to CH_4_ possible. The size of this window depends on the thermodynamics of the actual H_2_-producing and H_2_-consuming physiologies (Zinder, 1993; Dolfing, 2013).

The thresholds of H_2_ consumption increase with increasing temperature in a way that is characteristic for the underlying process, i.e., differently for sulfate reduction < methanogenesis < homoacetogenesis (Conrad and Wetter, 1990; Hoehler et al., 1998; Fey and Conrad, 2000; Kotsyurbenko et al., 2001). The thresholds of syntrophic H_2_ production also increase with increasing temperature. However, syntrophy between H_2_ production and H_2_ consumption is only possible if the threshold of production is higher than that of consumption (Figure 3). The resulting window of permissive H_2_ partial pressures gradually shifts to higher values when temperature increases (Westermann, 1996; Schink, 1997; Hattori, 2008). Alternatively to H_2_, syntrophy can also be maintained with formate acting as electron shuttle (Schink et al., 2017) or even with direct electron transfer (Shrestha and Rotaru, 2014; Li et al., 2015). Formate and H_2_/CO_2_ are largely at equilibrium and thus energetically equivalent in most methanogenic environments (Schink et al., 2017; Montag and Schink, 2018).

Acetate in methanogenic environments also displays a characteristic threshold that changes with temperature (Fey and Conrad, 2000). Like H_2_, acetate is an intermediate in the anaerobic degradation of organic matter to CH_4_. It is produced by many fermentation processes, notably by homoacetogenesis both from carbohydrates and from H_2_ + CO_2_ (Drake, 1994), and by many syntrophic degradation processes (Schink, 1997; McInerney et al., 2008). The syntrophic degradation processes can be subject to a decisive thermodynamic sensitivity for increased acetate concentrations, which may become inhibitory if too high (Ahring and Westermann, 1988; Dolfing and Tiedje, 1988; Beaty and McInerney, 1989; Platen et al., 1994). In methanogenic systems acetate is degraded to CH_4_ and CO_2_ by the genera *Methanosarcina* and *Methanothrix* only. Thermodynamics predict that the concentration of acetate, which is permissive for aceticlastic methanogenesis, decreases with temperature (ΔS^o^ > 0; Table 1), which indeed was observed in methanogenic paddy soil (Fey and Conrad, 2000). However, acetate concentrations are usually sufficiently high to allow exergonic CH_4_ production in methanogenic ecosystems (Klüber and Conrad, 1998; Yao and Conrad, 1999; Beer and Blodau, 2007). The decrease of acetate concentrations observed by Fey and Conrad (2000) was consistent with a change in the active methanogenic populations from a predominance of *Methanosarcina* to *Methanothrix* with relatively high versus low acetate thresholds (Jetten et al., 1992).

## Temperature dependence of carbon flow

Methane production from organic carbon follows characteristic degradation pathways that depend on which substrate the methanogenic archaea utilize. The major substrates of methanogenic archaea are acetate, H_2_ + CO_2_ and methyl compounds such as methanol, trimethyl amine or dimethyl sulfide. In most natural environments it is acetate and H_2_ + CO_2_ that dominate (Conrad, 1999; Conrad, 2020). The pathway of carbon flow providing acetate and H_2_ + CO_2_ from the degradation of organic matter may vary considerably between 100% acetate and 100% H_2_ + CO_2_ and any mixture in between (Conrad, 2020). Polysaccharides are an important class of organic matter. Cellulose and xylane are the major forms of dead plant material. Complete methanogenic degradation of cellulose occurs in four major steps, i.e., (1) hydrolysis and primary fermentation, (2) syntrophic secondary fermentation, (3) hydrogenotrophic methanogenesis, and (4) aceticlastic methanogenesis. Because of simplicity the following stoichiometries present lactate as sole product of primary fermentation. In methanogenic environments, other primary fermentation products, such as propionate, are actually more important. However, the principles of degradation are the same (Figure 1):

1: C_6_H_12_O_6_ → 2CH_3_CHOHCOOH

2: 2CH_3_CHOHCOOH + 2H_2_O → 2CH_3_COOH + 2CO_2_ + 4H_2_

3: 4H_2_ + CO_2_ → CH_4_ + 2H_2_O

4: 2CH_3_COOH → 2CH_4_ + 2CO_2_

Sum: C_6_H_12_O_2_ → 3CH_4_ + 3CO_2_

The pathway starts with primary fermentation (1) of sugar to lactic acid, followed by secondary syntrophic fermentation (2) of lactic acid to acetate, CO_2_ and H_2_. There are alternative pathways, e.g., via ethanol, butyrate or propionate, which all eventually result in the formation of 2 acetate, 2 CO_2_ and 4 H_2_. In such ‘syntrophic’ pathways aceticlastic and hydrogenotrophic methanogenesis contribute 67% (2 CH_4_) and 33% (1 CH_4_) to total CH_4_ production (3 CH_4_), respectively. The ‘syntrophic’ pathway is quite common for many methanogenic environments including anaerobic digestors (Zehnder, 1978; McCarty and Smith, 1986), rice field soils (Conrad and Frenzel, 2002; Conrad, 2007), peat bogs (Drake et al., 2009; Lai, 2009) and lake sediment (Ward and Winfrey, 1985; Capone and Kiene, 1988), especially at moderate temperatures (20–35°C).

Many studies have shown that the anaerobic degradation pathway can change with the *in-situ* temperature (Conrad, 2020). At low temperatures, in particular, the contribution of acetate can increase to 100%. This may happen when hydrogenotrophic methanogenesis (3) is replaced by with chemolithotrophic homoacetogenesis (5) (Figure 1):

1: C_6_H_12_O_6_ → 2CH_3_CHOHCOOH

2: 2CH_3_CHOHCOOH + 2H_2_O → 2CH_3_COOH + 2CO_2_ + 4H_2_

5: 4H_2_ + 2CO_2_ → CH_3_COOH + 2H_2_O

4: 3CH_3_COOH → 3CH_4_ + 3CO_2_

Sum: C_6_H_12_O_2_ → 3CH_4_ + 3CO_2_

Or it may happen when sugar fermentation to lactic acid (1) is replaced by homoacetogenic sugar fermentation (6):

6: C_6_H_12_O_6_ → 3CH_3_COOH

4: CH_3_COOH → 3CH_4_ + 3CO_2_

Sum: C_6_H_12_O_2_ → 3CH_4_ + 3CO_2_

It has been shown for many anoxic environments that homoacetogenesis is a favored process at low (<15°C) temperatures (Conrad et al., 1989; Nozhevnikova et al., 1994), so that hydrogenotrophic methanogenesis decreases relative to aceticlastic methanogenesis. A dominance of aceticlastic methanogenesis at low temperatures has been observed in rice paddy fields (Chin and Conrad, 1995; Fey and Conrad, 2000; Fu et al., 2018), lake sediments (Schulz and Conrad, 1996; Nozhevnikova et al., 1997; Glissmann et al., 2004) and soils (Küsel and Drake, 1995; Fu et al., 2015). However, it has not been observed in anaerobic marine sediments (Roussel et al., 2015) and in many boreal and arctic peat bogs (Kotsyurbenko et al., 2004; Metje and Frenzel, 2007; Tveit et al., 2015). In an Alaskan bog, CH_4_ is not produced from acetate, although it is the dominant product of organic matter degradation. Instead, it is only degraded by oxic respiration and ferric iron reduction (Duddleston et al., 2002). Hence, although acetogenesis and acetoclastic methanogenesis seems to be enhanced at low temperatures, the degradation of organic matter via both acetate and H_2_ + CO_2_ is not excluded.

Also at high temperature (>45°C), aceticlastic and hydrogenotrophic methanogenesis can operate producing 67 and 33% of the CH_4_, respectively, as observed in anaerobic digestors (Zinder et al., 1984; Vanlier et al., 1993; Gehring et al., 2015) and in rice field soils (Liu et al., 2018). This is the case, since thermophilic or thermotolerant microbes exist, which act as syntrophic secondary fermenters, as aceticlastic methanogens and as hydrogenotrophic methanogens (Vanlier et al., 1996; Imachi et al., 2000). Frequently, however, the contribution of H_2_ + CO_2_ increases at high temperatures to 100%, as acetate is consumed by syntrophic acetate oxidation (7) coupled to hydrogenotrophic methanogenesis (3) (Figure 1):

1: C_6_H_12_O_6_ → 2CH_3_CHOHCOOH

2: 2CH_3_CHOHCOOH + 2H_2_O → 2CH_3_COOH + 2CO_2_ + 4H_2_

7: 2CH_3_COOH + 4H_2_O → 8H_2_ + 4CO_2_

3: 12H_2_ + 3CO_2_ → 3CH_4_ + 6H_2_O

Sum: C_6_H_12_O_2_ → 3CH_4_ + 3CO_2_

The existence of syntrophic acetate oxidizing bacteria (7) and their general preference for high temperatures has been shown in anaerobic sludge digestors (Lee and Zinder, 1988a; Ahring, 1995; Hattori, 2008; Tang et al., 2008; Ho et al., 2013) and rice paddy fields (Fey et al., 2001; Conrad et al., 2009; Liu and Conrad, 2010; Rui et al., 2011; Liu et al., 2018). Evidence for syntrophic acetate oxidation at elevated temperature has also been found in an alkaline wetland soil in Tibet (Deng et al., 2019).

**Figure 4:**
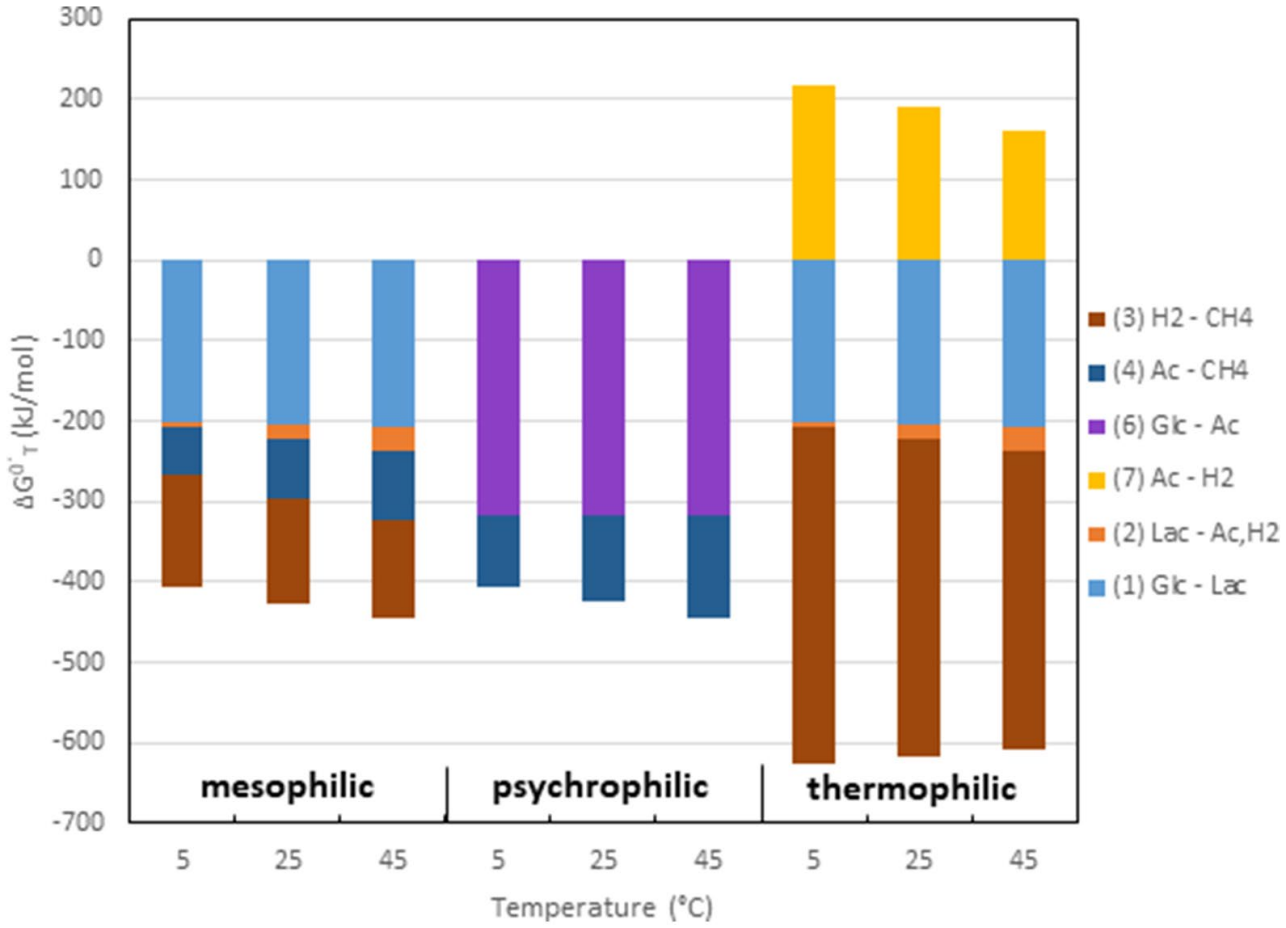
Values of ΔG^0^’_T_ at 5, 25, and 45°C of the different processes shown in Figure 1 and listed in Table 1. The processes are combined for typical reaction chains of mesophilic, psychrophilic and thermophilic degradation of organic matter to CH_4_ + CO_2_.

Although empirical studies have frequently shown that environments at intermediate temperatures (mesophilic) are dominated by the ‘syntrophic’ pathway (67% acetate, 33% H_2_/CO_2_), low temperatures (psychrophilic) by the acetogenic pathway (<67% acetate), and high temperatures (thermophilic) by the hydrogenotrophic pathway (100% H_2_/CO_2_), such preferences are by no means obligatory. The mechanistic reasons for these preferences are not quite clear. A look at the thermodynamics of the degradation pathways may be helpful. The values of ΔG^o^ are constrained by the stoichiometries of the pathways. The values change with temperature according to the magnitude of ΔS^o^ (Table 1). Therefore, the ΔG^o^_T_ values of the different degradation steps also change with temperature. The ΔG^o^_T_ of the entire process (cellulose → 3CH_4_ + 3CO_2_) is always −405, −426, and −445 kJ at 5, 25, and 45°C, thus moderately decreasing (becoming more exergonic) with temperature. The ΔG^o^’_T_ values of the individual reactions at 5, 25, and 45°C are shown in Figure 4. The data demonstrate that syntrophic secondary fermentation reactions producing H_2_ become increasingly less endergonic or more exergonic, if temperature increases. Figure 4 only shows the syntrophic degradation of lactate and acetate, but that of ethanol, butyrate, and propionate would be in-between. As a consequence, thermodynamics suggest that hydrogenotrophic and ‘syntrophic’ pathways are favored at increasing temperature. Since the acetogenic pathway from sugars (reaction 6 in Figure 1) is thermodynamically feasible at every temperature (small ΔS^o^, Table 1) this may be the reason why it predominates at low temperatures where the other degradation pathways are thermodynamically restricted. Although homoacetogenesis from H_2_ + CO_2_ is thermodynamically less favorable than hydrogenotrophic methanogenesis, and results in relatively higher H_2_ thresholds (Figure 3), the former can possibly prevail since psychrophilic homoacetogens seem to exist in many environments while psychrophilic methanogens are missing (Conrad et al., 1989; Kotsyurbenko et al., 2001). In addition homoacetogens can operate in a mixotrophic way. Homoacetogenic bacteria are able to use many different substrates. Therefore, thresholds for H_2_ can be lower with two substrates than with H_2_ alone (Peters et al., 1998).

However, the dominance of the hydrogenotrophic pathway at high temperatures requires different explanations. In fact, syntrophic degradation of lactate (or ethanol, butyrate, propionate) and aceticlastic methanogenesis both become thermodynamically more favorable when temperature increases (Figure 4). Thus, why should aceticlastic methanogenesis be replaced by syntrophic acetate oxidation and hydrogenotrophic methanogenesis? The minimum threshold concentration of acetate can be calculated from the respective ΔG^o^_T_ values using he Nernst equation. However, the ΔG^o^_T_ values are the same independently by which mechanism the conversion of acetate is achieved and so are the minimum thresholds of acetate. At the time being, one can only speculate why syntrophic acetate oxidation is dominant at high temperature. One possibility is that cell integrity of aceticlastic methanogens is worse at high temperatures than that of hydrogenotrophic methanogens. However, thermophilic species do exist for both aceticlastic genera *Methanothrix* and *Methanosarcina* (Zinder and Mah, 1979; Zinder et al., 1987; Kamagata et al., 1992; Mladenovska and Ahring, 2000). Another possibility is that it is preferable that the available energy is shared by two rather than one organism. Such sharing is not uncommon, e.g., oxidation of ammonia to nitrate by either one (comammox; Daims et al., 2015) or more commonly by two bacterial species (e.g., *Nitrosomonas*, *Nitrobacter*). Sharing reduces the length of the pathway (number of enzymatic steps) for the individual species, which may be favorable for energetic reasons (Costa et al., 2006). Since thermodynamics become more favorable at increasing temperatures, the syntrophic sharing option may become more attractive.

In fact, energy sharing among different physiological groups seems to be common for anaerobic breakdown of organic matter, even for simple sugars. This is seen in the multiple syntrophic degradation processes (e.g., of lactate, ethanol, butyrate, propionate), in which energy is usually shared by at least three different microbes, (1) the secondary fermenters producing H_2_, acetate and CO_2_ as final products, (2) hydrogenotrophic methanogens and (3) aceticlastic methanogens (Schink, 1997). Three partners have to share the energy content of substrate degradation. It is amazing, that degradation is not simply achieved by a single microbe, i.e., a methanogen, which would convert the substrate to CH_4_ + CO_2_ + H_2_O without having to share the energy. However, such methanogens are unknown, probably since their evolution was not competitive against the shared mode of metabolism.

## Temperature dependence of microbial community composition

**Figure 5:**
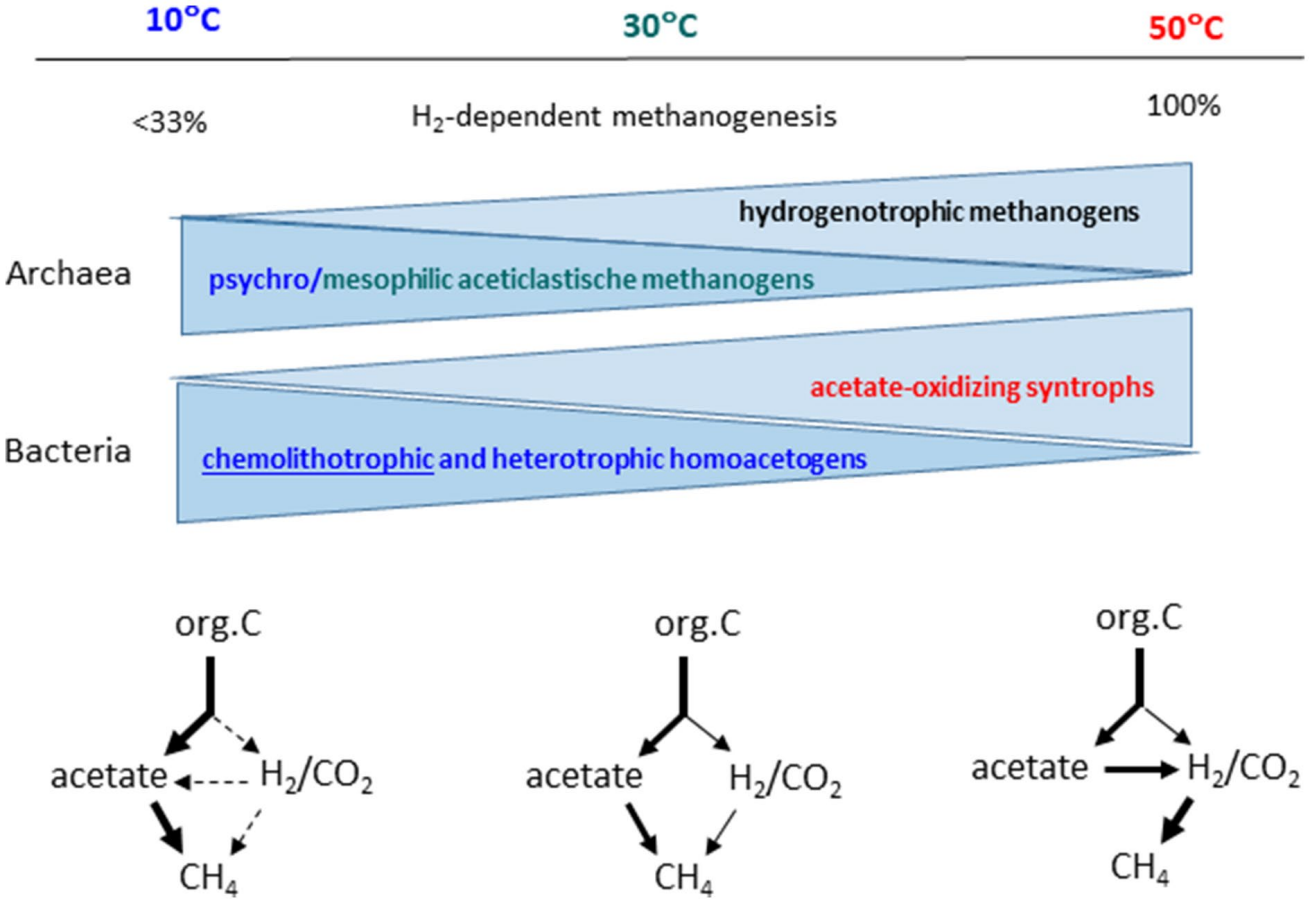
Relative contribution of hydrogenotrophic methanogenesis, composition of the microbial communities, and pathways of methanogenic degradation of organic matter as being characteristic for psychrophilic (10°C), mesophilic (30°C) and thermophilic (50°C) conditions.

The change of the pathway of organic matter degradation with increasing temperature makes it likely, that the microbial community responsible for the methanogenic degradation also changes. This is indeed the case. Many studies exist for rice field soils (Figure 5). Thus, the communities of methanogenic archaea are different at low, medium and elevated temperatures (Chin et al., 1999; Fey and Conrad, 2000; Wu et al., 2002; Rui et al., 2009; Peng et al., 2018; Liu et al., 2019). Most notable is the change from mesophilic to moderately thermophilic conditions, which shows a decrease of aceticlastic *Methanosarcina* and *Methanothrix* species and an increase of thermophilic *Methanocella* species (formerly RC-I), which are hydrogenotrophic (Conrad et al., 2009; Lu et al., 2015; Liu et al., 2018; Peng et al., 2018). Hence hydrogenotrophic methanogens are most likely involved in the syntrophic degradation of acetate under thermophilic conditions. The population size of putative syntrophic acetate oxidizers is also enhanced under thermophilic conditions, namely *Thermoanaerobacter* species relatively increase (Liu and Conrad, 2010; Rui et al., 2011; Liu et al., 2018; Peng et al., 2018). However, moderately thermophilic methanogens are ubiquitous in the environment, members of the hydrogenotrophic genus *Methanocella* (Fey et al., 2001), but also other hydrogenotrophic genera [e.g., *Methanobacterium*, *Methanoregula* (Peng et al., 2008; Deng et al., 2019)] and aceticlastic *Methanosarcina* and *Methanothrix* as well (Wu et al., 2006). Therefore, it is not surprising that rice fields exist in which thermophilic degradation of acetate is achieved by canonical aceticlastic methanogenesis (Liu et al., 2018). Nevertheless, even with aceticlastic methanogenesis the methanogenic microbial communities form a different network at high versus moderate temperatures (Peng et al., 2018; Liu et al., 2019).

Thermophilic syntrophic acetate conversion also has frequently been observed in anaerobic digestor systems usually fed with waste or waste water. The microbial communities under mesophilic versus thermophilic conditions are generally different. However, the taxa of putative thermophilic syntrophic acetate oxidizers are quite diverse in different digestors. Early enrichments resulted in an unnamed homoacetogenic bacterium (Lee and Zinder, 1988b) and in *Thermoacetogenium phaeum* (Hattori et al., 2000; Hattori, 2008). Later analyses of digestor communities using molecular tools indicate the operation of various taxa of syntrophic acetate oxidizers, such as *Thermotogae, Dethiobacteraceae, Clostridium, Hydrogenophaga, Fervidobacterium, Spirochaeta, Limnohabitans, Rhodococcus, Thermoacetogenium, Tepidiphilus, Petrobacter* (Leven et al., 2007; Tang et al., 2008; Hao et al., 2011; Sun et al., 2018; Dyksma et al., 2020). Thermophilic methanogens include the genera *Methanobacterium*, *Methanoculleus*, *Methanothermobacter*, and *Methanosarcina* (Leven et al., 2007; Tang et al., 2008; Hao et al., 2011; Ho et al., 2013; Dyksma et al., 2020). Note, however, that mesophilic syntrophic acetate oxidizers also exist, e.g., *Clostridium ultunense* (Schnürer et al., 1996) or *Syntrophaceticus schinkii* (Westerholm et al., 2010).

Psychrophilic conditions also affect the methanogenic microbial communities in rice paddy soils, but the effects are more delicate than the difference between mesophilic and thermophilic conditions. Usually, homoactogenic activities are enhanced at low temperature (Figure 5). The acetogenic populations seem to belong to the *Clostridium* cluster I and *Peptococcaceae* (Liu and Conrad, 2011). Enhanced acetate production at low temperature is followed by consumption by aceticlastic methanogens of the genera *Methanosarcina* and *Methanothrix* (Chin et al., 1999; Wu et al., 2002; Peng et al., 2008). The overall diversity of methanogenic archaea seems to increase at low temperature, *Methanothrix* in particular (Chin et al., 1999; Wu et al., 2002).

Different microbial communities at psychrophilic versus mesophilic conditions were also observed in peat bogs and arctic wetlands (Hoej et al., 2008; Blake et al., 2015; Schmidt et al., 2015, 2016; Kolton et al., 2019). The taxa *Methanosarcina*, *Methanothrix*, *Methanobacteriaceae*, *Methanoregulaceae*, and *Methanocella* are generally common, but the aceticlastic genera (*Methanosarcina*, *Methanothrix*) are especially abundant at low temperatures. These taxa have also been found in a boreal mire, but with little changes in community composition over a seasonal temperature gradient between 0 and 14°C (Juottonen et al., 2008). The bacterial communities in peat lands have been found to be dominated by *Clostridiaceae* (Kolton et al., 2019) and by *Pelobacter*, *Syntrophobacteraceae*, *Syntrophaceae* and *Syntrophorhabdaceae* as potential secondary fermenters (Schmidt et al., 2015, 2016).

## Conclusion

Methanogenic microbial communities catalyze the anaerobic degradation of organic matter to CO_2_ and CH_4_. This process is basically the same in various environments, such as rice paddy fields, wetlands, lake sediments, peat bogs, anaerobic digestors, or the intestinal tract of animals. The pathway of the process is also basically the same. Irrespective of the particular environment, the degradation steps and the metabolic classes of the microrganisms involved are basically the same (Figure 1). Nevertheless, the exact composition of the methanogenic microbial communities can be quite different in the different environments. Furthermore, the composition can change with temperature, thus resulting in a change of the pathway of the degradation process. Thus, it is frequently observed that the pathway and the responsible methanogenic microbial community changes from psychrophilic to mesophilic to thermophilic conditions, with dominance of aceticlastic methanogenesis at low and hydrogenotrophic methanogenesis at high temperatures (Figure 5). Such behavior is consistent with thermodynamics of the critical steps in organic matter degradation, but is nevertheless not obligatory. Thus, environments exist, in which CH_4_ production is dominated by hydrogenotrophic methanogenesis despite low temperatures and aceticlastic methanogenesis despite high temperatures, simply because these environments contain the respective psychrophilic and thermophilic species.

Microorganisms proliferate within and tolerate a more or less wide range of temperatures. This is observed in environments that experience only small temperature fluctuations (lake sediments, technical digesters, hot springs) and also in others that experience dramatic temperature changes on a daily or seasonal range (littoral sediments, rice paddies, peatlands). The microorganisms generally display characteristic temperature optima and a characteristic increase of reaction kinetics with increasing temperature, which can be modelled by the Arrhenius equation using a characteristic apparent activation energy (*E_a_*). Interestingly, methanogenic archaea were found to exhibit a rather high range of *E_a_* values of >100 kJ mol^−1^. In the environment, hydrolysis of complex organic matter is the first step of the methanogenic degradation processes, and this process can exhibit markedly lower *E_a_* values of about 60 kJ mol^−1^. However, analysis of various methanogenic environments with complex microbial communities display *E_a_* values, which are not similar to those of hydrolysis but to those of the methanogenic archaea. This observation indicates that hydrolysis of organic matter is not the rate limiting step of CH_4_ production in most environments, meaning that the microbial community is frequently supplied with pulses of easily degradable substrates.

## Author contributions

The author confirms being the sole contributor of this work and has approved it for publication.
